# 681. Antibiotic Treatment During Hospitalization Is a Major Risk Factor for The Occurrence Of *Clostridium Difficile* Infection Among Carriers

**DOI:** 10.1093/ofid/ofad500.743

**Published:** 2023-11-27

**Authors:** Mayan Gilboa, Ido Cohen, Yovel Peretz, Eyal Meltzer, Dafna Yahav, Gili Regev-Yochay

**Affiliations:** Sheba Medical Center, Aventura, Florida; Sheba Medical Center, Aventura, Florida; Sheba Medical Center, Aventura, Florida; Sheba Medical Center, Aventura, Florida; Sheba Medical Center, Aventura, Florida; Sheba Medical Center, Aventura, Florida

## Abstract

**Background:**

Asymptomatic carriers of *Clostridium difficile (CD)* have a higher risk of developing CD infection (CDI). Previous studies have shown that reducing inappropriate antimicrobial use through antimicrobial stewardship programs can decrease rates of CDI. However, risk factors for the development of CDI among carriers, particularly the role of antibiotic treatment, have not been well-defined.

Risk factors for Clostridium Difficile Infection - multivariable logistic regression analysis

**Figure:**
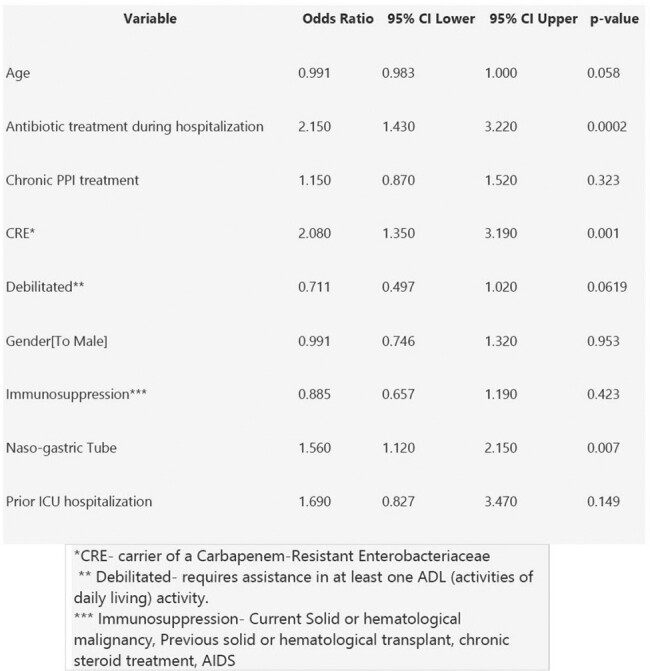

**Methods:**

We conducted a prospective cohort study between January 2017 and March 2023, assessing 2524 asymptomatic CD carriers admitted to internal medicine and hematology wards. Patients were screened for CD carriage upon admission and followed untill discharge for occurence of symptomatic CDI. We performed multivariable logistic regression analyses adjusted for age, gender, immunosuppression status, Carbapenem-resistant Enterobacteriaceae (CRE) carriage, Previous intensive care unit admission in the past 30 days, Presence of a nasogastric tube (NG), chronic Protein Pump Inhibitor (PPI) usage, and debilitation and examined whether at least one antibiotic dose, either Intra venous or oral, during the hospitalization, was related to the occurrence of clinical CDI.

**Results:**

Of the 2524 CD carriers, 225 (8.9%) developed clinical CDI during their hospitalization. In a multivariable logistic regression analysis, antibiotic treatment during hospitalization was the most significant risk factor for the occurrence of CDI among carriers (OR 2.15, 95% CI 1.43-3.22). Other risk factors included CRE carriage (OR 2.08, 95% CI 1.35-3.19) and the presence of an NG tube (OR 1.56, 95% CI 1.12-2.15).

**Conclusion:**

In this large prospective cohort study, we found that antibiotic treatment during hospitalization was the most significant risk factor for the occurrence of CDI among carriers. These results emphasize the importance of antimicrobial stewardship programs and suggest that CD carriers represent a suitable target for focused stewardship efforts

**Disclosures:**

**All Authors**: No reported disclosures

